# Supplementary material for: Polyphenol Compound 18a Modulates UCP1-Dependent Thermogenesis to Counteract Obesity
Source: Biomolecules. 2024 May 23;14(6):618. doi: 10.3390/biom14060618 (PMC11201655; doi:10.3390/biom14060618)
Supplement: Supplementary file 1 [file biomolecules-14-00618-s001.zip › biomolecules-2996814-supplementary.pdf]

Supplementary Table S1. Primer sequences used in real time Q-PCR (mouse).

Genes	Sequence
36b4 forward	TTTGGGCATCACCACGAAAA
36b4 reverse	GGACACCCTCCAGAAAGCGA
Prkaa1 forward	AAAGTGAAGGTGGGCAAGCA
Prkaa1 reverse	CAGATGGTGTACTGATGACCTGG
Prkaa2 forward	TCGCAGTTTAGATGTTGTTGGA
Prkaa2 reverse	CTTCAACCCGCCCATGTTTG
Ucp1 forward	ACTGCCACACCTCCAGTCATT
Ucp1 reverse	CTTTGCCTCACTCAGGATTGG
Pgc1α forward	ACTGAGCTACCCTTGGGATG
Pgc1α reverse	TAAGAATTTCGGTGGTGACA
Prdm16 forward	CAGCACGGTGAAGCCATTC
Prdm16 reverse	GCGTGCATCCGCTTGTG
Cox7a1 forward	CAGCGTCATGGTCAGTCTGT
Cox7a1 reverse	AGAAAACCGTGTGGCAGAGA
Pparα forward	AGGCCGTTGCCACTGTTCAG
Pparα reverse	AGCCCTCTTCATCCCCAAGC
Cidea forward	TGCTCTTCTGTATCGCCCAGT
Cidea reverse	GCCGTGTTAAGGAATCTGCTG
Cox8b forward	GAACCATGAAGCCAACGACT
Cox8b reverse	GCGAAGTTCACAGTGGTTCC
Dio2 forward	AATTATGCCTCGGAGAAGACCG
Dio2 reverse	GGCAGTTGCCTAGTGAAAGGT
Errα forward	GCAGGGCAGTGGGAAGCTA
Errα reverse	CCTCTTGAAGAAGGCTTTGCA
Atp2a1 forward	TTCATCCGCTACCTCATCT
Atp2a1 reverse	CCATCAGTCACCAAGTTCA
Atp2a2 forward	GCATGCACCGATGGCATTTC
Atp2a2 reverse	ATGTAGCGCTGTCAGTCAAGA
Ryr1 forward	TCTTCCCTGCTGGAGACTGT
Ryr1 reverse	GTGGAGAAGGCACTTGAGG
Ryr2 forward	ATGGCTTTAAGGCACAGCG
Ryr2 reverse	CAGAGCCCGAATCATCCAGC
Camk2 forward	AACTGACCAGGCACAGACG
Camk2 reverse	CCCTAATGTCTTCCGCCTGC
Serca2 forward	TCGACAGGACAGAAAGAGTGTG
Serca2 reverse	AAACTGAATTCAACTCACCAGC

Supplementary Table S2. Compound structure.

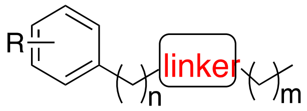

Compound	Linker	R	m	n
8i	cis-ethylene-1,2-diyl	3-OMe-5-OMe	5	6
8j	cis-ethylene-1,2-diyl	3-OH-5-OH-6-Cl	5	6
18a	m-phenylene	3-OH-5-OH	5	3
18f	m-phenylene	3-OH-5-OH	5	1
18i	m-phenylene	3-OH-5-OH	5	7
18k	m-phenylene	3-OH-5-OH	4	3
18l	m-phenylene	3-OH-5-OH	6	3
18o	m-phenylene	3-OH-4-F-5-OH	5	3
18p	m-phenylene	3-OH-5-OH-6-Cl	5	3

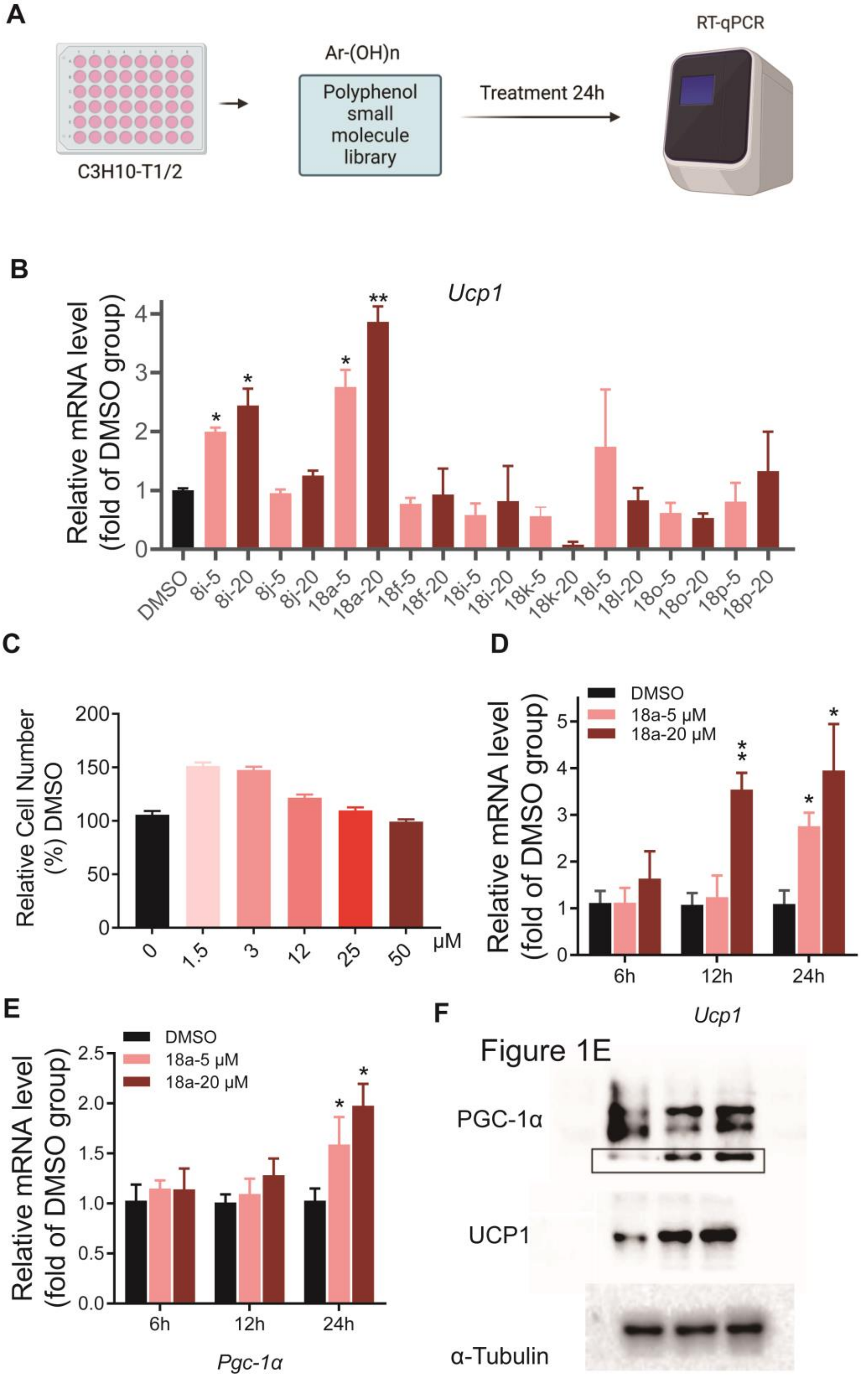
A
C3H10-T1/2
Ar-(OH)n
Polyphenol small molecule library
Treatment 24h
RT-qPCR
B
Ucp1
Relative mRNA level (fold of DMSO group)
DMSO
8i-5
8i-20
8j-5
8j-20
18a-5
18a-20
18f-5
18f-20
18i-5
18i-20
18k-5
18k-20
18l-5
18l-20
18o-5
18o-20
18p-5
18p-20
C
Relative Cell Number (%) DMSO
μM
D
DMSO
18a-5 μM
18a-20 μM
Relative mRNA level (fold of DMSO group)
6h
12h
24h
E
DMSO
18a-5 μM
18a-20 μM
Relative mRNA level (fold of DMSO group)
6h
12h
24h
Pgc-1α
F
Ucp1
Figure 1E
PGC-1α
UCP1
α-Tubulin

Supplementary Figure S1 18a activates UCP1 expression in adipocytes and exhibits a satisfactory safety profile.

A. Screening Flowchart; B. Transcriptional screening data (compound structures are in Table 2); C. Cell vitality after different concentration of 18a; D. Transcriptional screening data of *Ucp1*; E. Transcriptional screening data of *Pgc1α*. F. Original western blot images. n = 4 per group. Data presented as the means ± SEM. *p < 0.05, **p < 0.01 compared with the indicated control group.

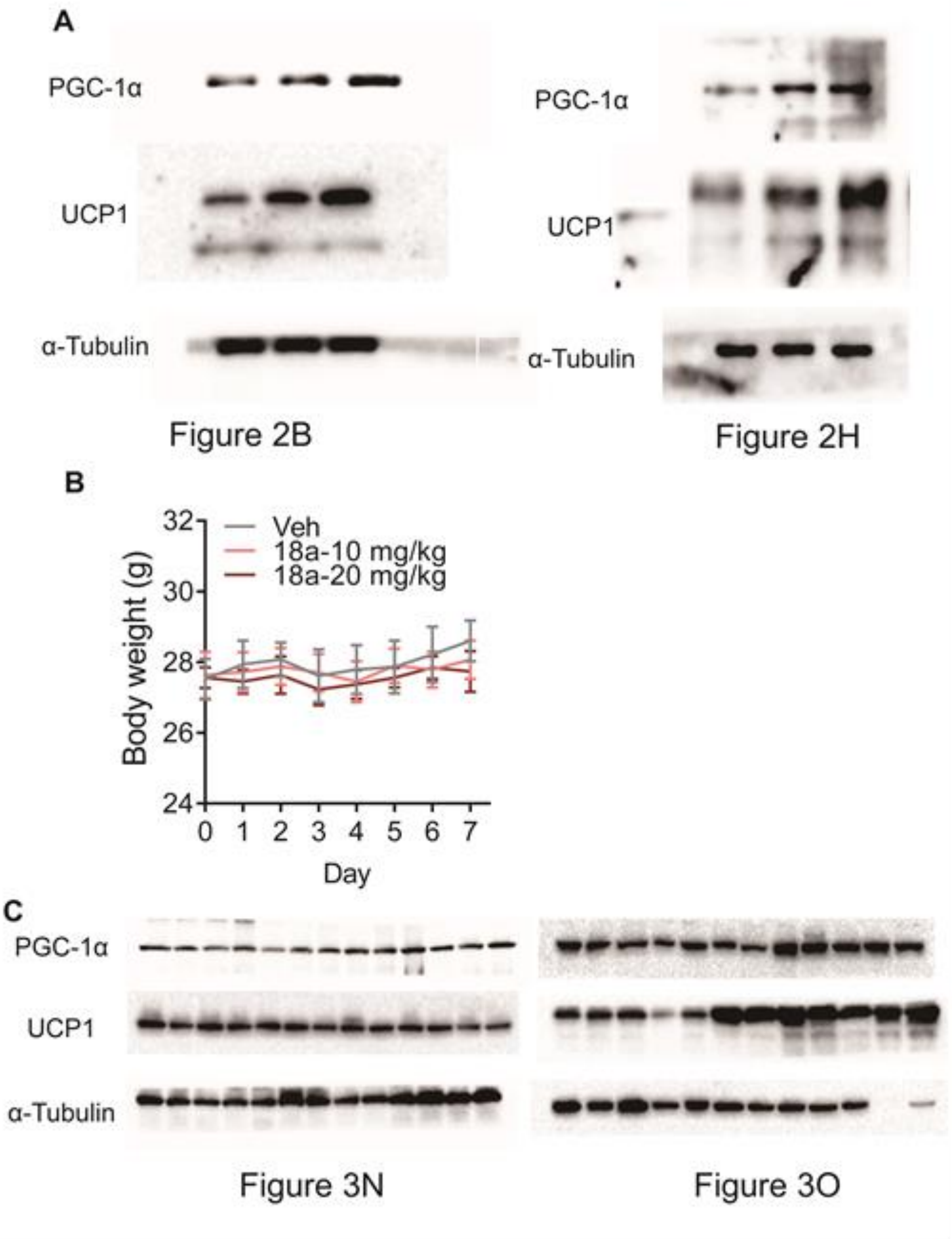

Supplementary Figure S2 There was a tendency to reduce body weight in mice when short-term 18a treatment.

A&C. Original western blot images. B. Body weight changes in FVB mice. n = 6 per

group. Data presented as the means ± SEM.

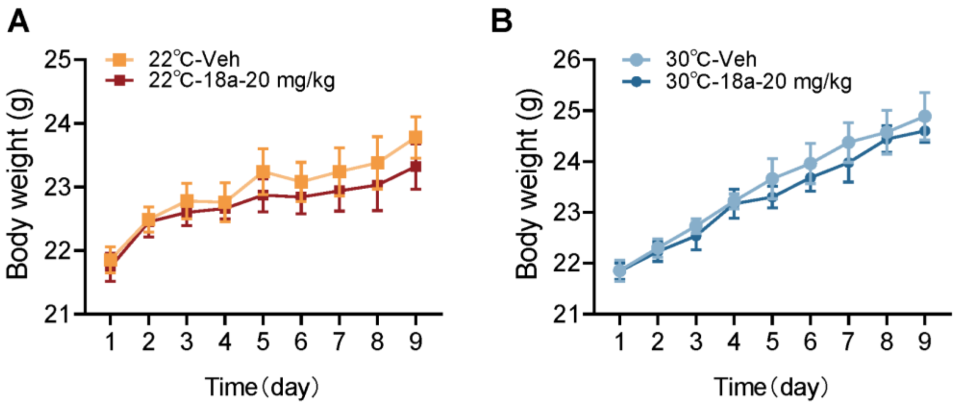

Supplementary Figure S3 Body weight changes in mice administered with 18a at 22°C and 30°C.

A. Body weight changes in mice administered with 18a at 22°C; B. Body weight changes in mice administered with 18a at 30°C. n = 6 per group. Data presented as the means ± SEM.

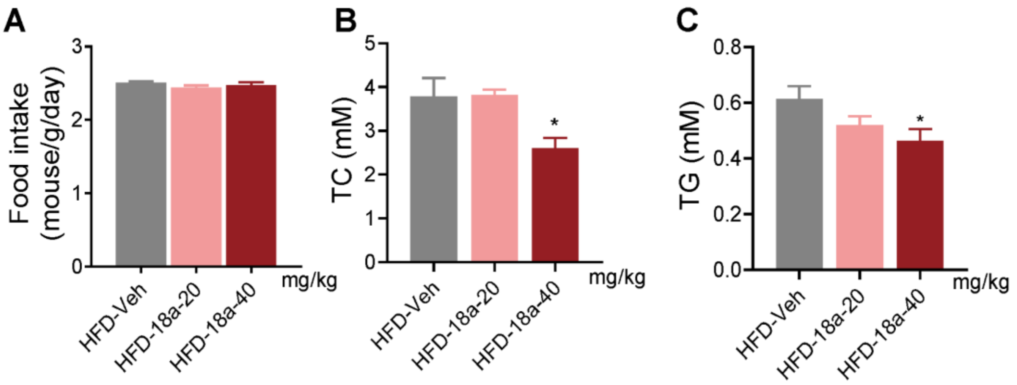

Supplementary Figure S4 18a reduces plasma lipid accumulation in high-fat-induced mice without altering food intake.

A. Average food intake; B-E. Plasma concentrations of total cholesterol (TC), triglyceride (TG). n = 6 per group. Data presented as the means ± SEM. *$p < 0.05$ compared with the HFD-Vehicle group.

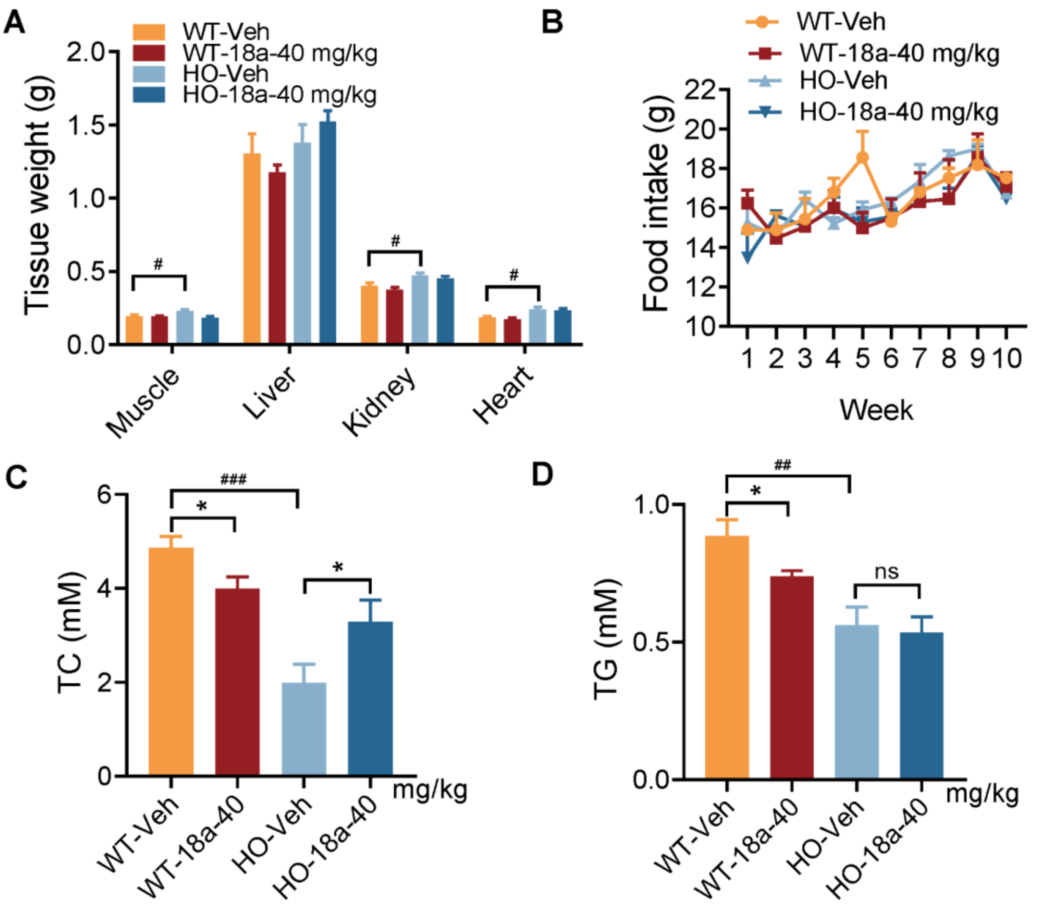

Supplementary Figure S5 18a reduces plasma lipid accumulation in HFD induced mice without altering food intake, but is not effective in HO mice.

A. Tissue weight of WT&HO mice; B. Food intake; C-D. Plasma concentrations of total cholesterol (TC), triglyceride (TG). n = 6 per group. Data presented as the means ± SEM. *$p < 0.05$ compared with the Vehicle group. $^{\#}p < 0.05$, $^{\#\#}p < 0.01$, $^{\#\#\#}p < 0.01$, WT group versus HO group.

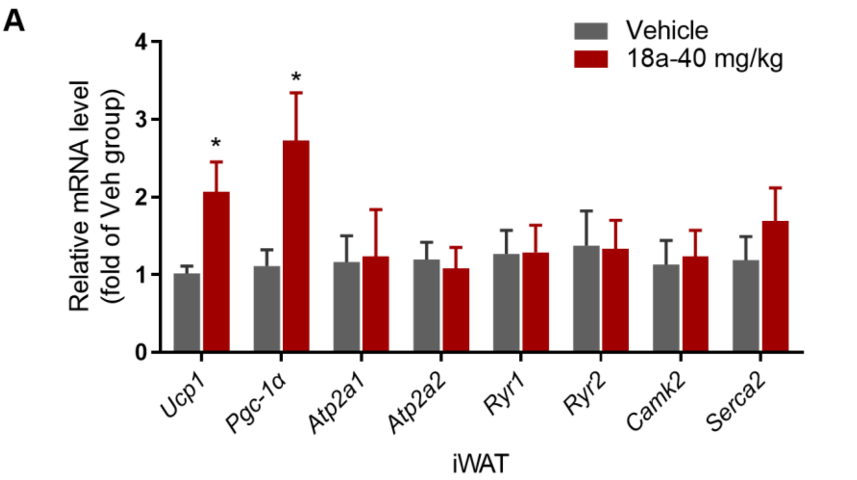

Supplementary Figure S6. 18a does not affect UCP1 independent thermogenesis in iWAT.

A. Expression of the UCP1 independent thermogenic gene in iWAT of HFD mice treated 18a. n = 6 per group. Data presented as the means ± SEM. *p < 0.05 compared with Vehicle group.

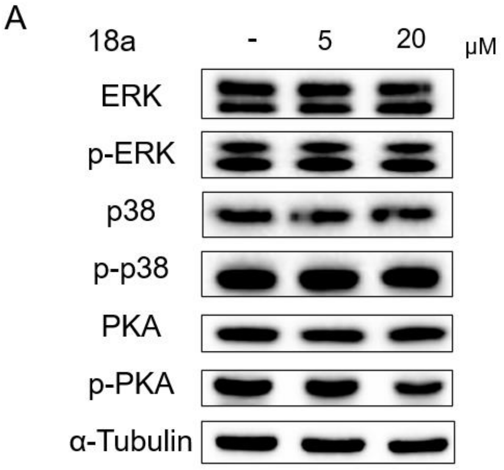

Supplementary Figure S7. Changes in thermogenesis-related signaling pathway protein levels following 1-hour treatment with 18a

A. Protein level detection in C3H10-T1/2.

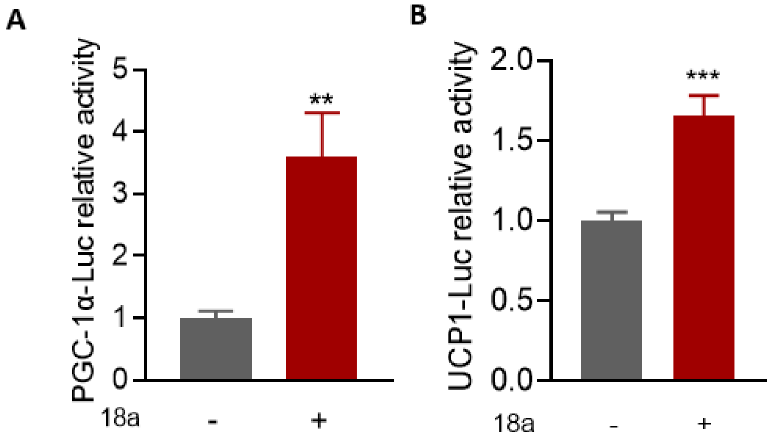

Supplementary Figure S8. 18a enhances the transcriptional activity of PGC-1α and UCP1 promoter.

A. HEK293 cells were transfected with PGC-1α-Luc plasmid, treated with 18a for 24 hours, and fluorescent signals were collected; B. HEK293 cells were transfected with UCP1-Luc plasmid, treated with 18a for 24 hours, and fluorescent signals were collected; n = 6 per group. Data presented as the means ± SEM. *p < 0.05 compared with DMSO group.

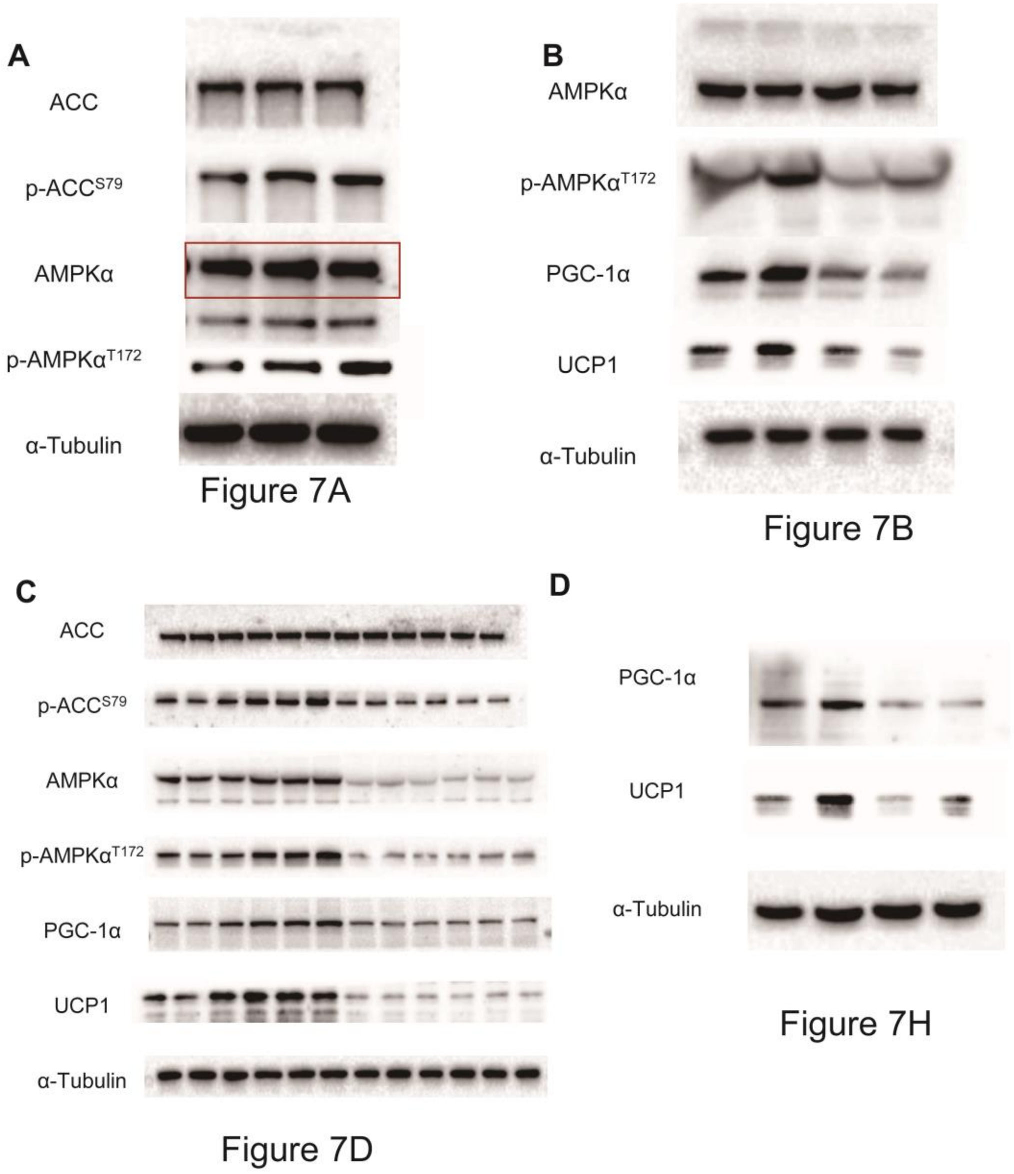

Supplementary Figure S9. Original western blot images.
